# Antiphlogistine versus Opium

**Published:** 1907-09

**Authors:** 


### ANTIPHLOGISTINE VERSUS OPIUM.

Inflamed states of the various organs of the body frequently give rise to pain of such urgent character as to demand active steps looking to its relief. Upon seeing the patient for the first time (he has called his physician because his suffering has become intolerable), the medical attendant is met with a peremptory demand for relief from the suffering.

With a willingness which frequently overrides their better judgment, some physicians resort to the hypodermic needle indiscriminately, and, in too many cases, a greater evil has followed the lesser one. The free habit of using morphine or some other form of opium is not a judicious practice, and for several reasons. The exact seat of an inflammation for instance, might become difficult to locate, and thus a clear diagnosis interfered with. But the greater objection to the use of opium is the possibility of adding a recruit to the ever growing army of habitues.

Every time there occurs to a doctor the apparent need for opium he should deliberate well before resort is had to the needle. If, after careful consideratson, his best judgment advises the use of opium, it should be given in some form by mouth. If the needle is used the patient at once knows what he is getting, but he is not so likely to acquire this information if it be given otherwise.

For relieving the pain of the inflammations Antiphlogistine will easily take the place of opium. The relief following may not be so prompt and so complete, but the edge of the suffering is taken off within a short time, and soon the patient is in a comfortable condition and has escaped the possibility of becoming addicted to a drug. There is not the likelihood that a patient, relieved from pain by it, will begin eating or using Antiphlogistine in any other way, which likelihood is the greatest disadvantage of opium.

In the future let your morphine become stale, and keep your Antiphlogistine fresh—use it in inflammation.—The Medical Era.

